# Calculating punitive damage multiplier in intellectual property cases: An empirical study and the enhanced model

**DOI:** 10.1371/journal.pone.0308447

**Published:** 2025-02-21

**Authors:** Jiangang Shang, Weiqi Xu, Weina Xu, Keyao Wu

**Affiliations:** 1 School of Economic Law, Shanghai University of Political Science and Law, Shanghai, China; 2 Shanghai Cancer Center and Shanghai Medical College, Fudan University, Shanghai, China; 3 School of Economics and Management, Shanghai University of Political Science and Law, Shanghai, China; 4 Department of Statistics, University of Chicago, Chicago, Illinois, United States of America; Far Eastern University - Manila, PHILIPPINES

## Abstract

The application of punitive damages in intellectual property cases in China has encountered notable deficiencies within the judicial context. These deficiencies, including a limited range of applicability, frequent recourse to statutory damages, difficulties in ascertaining the appropriate damages base, and a marked reliance on subjective factors in calculating multipliers, undermine judicial consistency and fairness. In this study, we conducted a comprehensive quantitative analysis by selecting 79 pertinent judgments from a dataset of 3,478 intellectual property rulings. Initially, we developed a multiple linear regression model to assess punitive damages. Upon encountering negative estimated coefficients, we improved the model to a Beta Generalized Linear Model. The efficacy of the new model was validated through an improved R-squared value compared to the original model and by analyzing an additional 45 cases. Both models highlight the scope of infringement and the reputation of intellectual property assets as critical variables. They suggest that wider infringements lead to greater economic penalties and emphasize the significant market value and potential financial losses of intellectual property. Significantly, this study represents a pioneering initiative in China, establishing a model to assist in the legal determination of punitive damages in IP cases.

## 1. Introduction

Determining the compensation for intellectual property (IP) infringement poses a challenge of global significance [1]. The punitive damages system represents the sixth method of calculating damages in China’s IP compensation framework, succeeding "the plaintiff’s losses," "the defendant’s profits from infringement," "referenceable license fees," "statutory damages," and "discretionary compensation." It stands as an intermediary mechanism between "civil law" and "criminal law," akin to a "quasi-criminal penalty." On January 1, 2021, the Civil Code of the People’s Republic of China (Civil Code) specifically outlined comprehensive provisions regarding punitive damages for IP infringement within the chapter on tort liability. This development marked the full implementation of the punitive damages system in intellectual property.

Following that, Article 6 of the "Interpretation of the Supreme People’s Court on the Application of Punitive Damages in the Trial of Civil Cases of IP Infringement" (Judicial Interpretation) was implemented on March 3, 2021, which stipulates that "when the people’s court determines the punitive damages per the law, it shall comprehensively consider the degree of subjective fault of the defendant, the severity of the infringement and other factors (Supreme People’s Court, 2021). In the same year, Chinese courts applied punitive damages to infringers in 895 IP cases (Supreme People’s Court, 2022). Punitive damages have at least three advantages. First, when criminal sanctions in IP are inconveniently implemented, punitive damages should be used as an alternative measure to criminal penalties to solve the judicial shortcomings of insufficient IP protection [2]. Second, punitive damages are a necessary supplement and improvement to the form of liability for infringement compensation, and they are the improvement of the IP [3]. Third, punitive damages contribute to the quantitative determination of damages. The most intuitive manifestation of solving quantitative problems is to increase compensation and innovate the compensation, of which punitive damage is an important part [4].

Although punitive damages have multiple functions, such as compensating the losses suffered by the victim and punishing and curbing wrongful acts, there are application and theoretical dilemmas. On the one hand, punitive damages currently face problems such as vague standards, excessive subjectivity, and the implementation of statutory damages instead of punitive damages [5]. On the other hand, there are disputes over different standards in the application process for punitive damages [6]. Punitive damages cases lack objective and unified multiple determination rules, so the determination of penalty cannot be explained in judicial practice, thus failing to provide parties with relatively stable judicial expectations [7]. With the increasing demand for and protection of IP, the focus on strengthening IP protection has changed from qualitative to quantitative.

Existing research only conducts a qualitative analysis of influencing factors from a subjective perspective, such as observing the "actual multiplier." No scholar has yet conducted a quantitative study on the contribution of various factors to punitive damages. We establish an effective punitive damages model to explore how to handle the tension between the legislation and practice correctly. We aim to clarify the applicable punitive damages model and solve the application problem of punitive damages to a certain extent.

## 2. Methodology

### 2.1 Refining factors impacting punitive damages multipliers

In China, determining punitive damages relies on two pivotal factors: "maliciousness" and "serious circumstances." Consequently, conducting a quantitative study on punitive damages necessitates meticulously refining specific indicators for these two factors. Given that punitive damages introduce a retaliatory element from criminal law into the predominantly restorative framework of civil law, caution should be exercised in selecting factors influencing punitive damages multipliers [8]. Existing research has explored functionally the factors that influence punitive damages. For instance, some scholars analyze from the perspective of "malice and intent," asserting that "maliciousness" embodies greater "subjective malignancy" than "intent." This is evident when the infringing party continues infringement after being warned by the opposing party after a court ruling instructing them to cease infringement or when there is an attempt to conceal the infringement [9]. Another analytical approach from scholars involves considering "malice and goodwill," contending that, compared with "intent," "maliciousness" carries the connotation of bad intentions. "Maliciousness" should entail evident moral responsibility, and it is further pointed out at an objective level that, although the damage may be minor, repeated infringements can still constitute "malicious intent" [10]. The term "serious circumstances" does not refer to a specific circumstance meeting a predefined standard but should entail an overall evaluation of the infringement of legal interests [11]. Simultaneously, "maliciousness" and "serious circumstances" are not isolated concepts but a comprehensive evaluative standard that combines subjective and objective elements. "Maliciousness" accentuates the blameworthiness of "serious circumstances," while "serious circumstances" expand the avenues for proving "maliciousness." The interdependence between these two factors is notable [12]. However, Chinese scholars have not clarified the specific index factors for "maliciousness" and "serious circumstances," nor have they quantified the index factors for these elements. Instead, many scholars express their assessment indicators collectively, using phrases such as "factors influencing punitive damages" [13]. The drawback of this approach lies in its inability to articulate the relative importance of different indicators.

Examining various countries globally, while the punitive damages system is currently assimilating into civil law, a distinct standard for the multiplier has not yet crystallized. Japan lacks a punitive damages system, and countries such as the United Kingdom, Australia, and Canada do not prescribe punitive damages, primarily relying on corresponding intellectual property and tort law rules for adjudication [14]. A common assertion among scholars is that the punitive damages system "solely exists in the United States." Nevertheless, Spain, France, and Italy recognize U.S. judgments on punitive damages, while Argentina, Brazil, Mexico, India, and Thailand have legislatively or substantively embraced punitive damages [15]. In addition to the United States, South Korea has codified punitive damages through legislation (PATENT ACT, 2022). It is apparent that, for most nations recently adopting the punitive damages system, determining the upper limit of punitive damages and the relevant factors represents an avant-garde legal exploration.

In the context of the United States, there is a notable absence of quantitative analysis in the calculation of punitive damages, leading to challenges such as the jury lacking a legal foundation to determine the appropriateness of punitive damages and the absence of established standards for quantifying the punitive damages amount [16]. The U.S. Patent Law exclusively addresses the criterion of "maliciousness" while neglecting to define "serious circumstances." It outlines the potential for increasing damages up to three times the initially determined or assessed amount, yet it fails to articulate specific rules or formulas for calculating the multiplier. When deliberating on punitive damages, local U.S. courts traditionally consider nine factors established in 1992. These factors include: 1) whether the infringer deliberately copied the ideas or design of another; 2) whether the infringer, when he knew of the other’s patent protection, investigated the scope of the patent and formed a good-faith belief that it was invalid or that it was not infringed; 3) the infringer’s behavior as a party to the litigation; 4) defendant’s size and financial condition; 5) closeness of the case; 6) duration of defendant’s conduct; 7) remedial action by defendant; 8) defendant’s motivation for harm; and 9) whether defendant attempted to conceal its misconduct (Fed. Cir., 1992). This judgment method persists today, albeit with some modifications (Fed. Cir., 2022). In the 2007 Seagate case, the judicial perspective evolved, perceiving the original method as setting a lower threshold for punitive damages.

Consequently, a "two-step test" was introduced, necessitating the patentee to demonstrate, with clear and convincing evidence, that the infringer’s behavior objectively indicates infringement of a valid patent. Subsequently, if this threshold is met, the patentee must further establish that the risk was known or so obvious that the alleged infringer should have been aware of it (Fed. Cir., 2022). However, this shift in the burden of proof from defendant to plaintiff has been critiqued for unduly complicating the plaintiff’s pursuit of applicable punitive damages. The Halo case reversed the decision in the Seagate case. The U.S. Federal Supreme Court asserted that the infringer’s intent encompasses both "intent" and "negligence," disregarding the consideration of "reckless conduct." The court allowed district courts to exercise discretion in imposing punitive damages and encouraged a case-by-case evaluation. An empirical analysis of punitive damages cases in the United States from 2010 to 2020 revealed that, exemplified by the Halo case, there was a 27.8% increase in the court’s determination of "intentional" compared to the period preceding Halo [17]. Despite the viewpoint of some scholars who argue that existing law does not mandate the necessity of "intent" for the application of punitive damages, suggesting the elimination of the "intent" requirement [18], the United States currently maintains the "intentional" standard as a prerequisite for applying punitive damages. Punitive damages are reserved for egregious cases characterized by intentional misconduct [19].

Foreign research has played a pivotal role in shaping China’s punitive damages system. However, given the unique characteristics of each legal system, the formulation of China’s punitive damages standards requires a grounding in local experience to establish the most suitable institutional framework. Applying quantitative jurisprudence mitigates the potential for ambiguous interpretations of legal texts by transforming texts and phenomena into symbols. Logical tools and techniques are then employed to deconstruct these texts. Judges Gong Xiaoyan and Liu Chang from the Shanghai Pudong New District Court have utilized the "factor accumulation method" to devise the "punitive Damage Influence Factor Table." This table encompasses various factors influencing punitive damages, with specified index ranges assigned to each factor [20]. An analysis has informed an update to this table of judicial cases.

The primary amendments and rationale for the update are delineated as follows: Firstly, the continuation of infringement by the infringer after the initial infringement judgment is deemed indicative of subjective malice. While analyzing the "maliciousness" element, the judges focused solely on scenarios where the plaintiff was subjected to administrative penalties and court injunctions. The "Judicial Interpretation" has now incorporated this behavior into "serious circumstances." Given that the plaintiff persists in infringement even after a conviction, malicious intent is evident. Secondly, the defendant’s deliberate implementation of piracy and counterfeiting of registered trademarks is identified as a subjective malicious situation in the "Judicial Interpretation." Hence, the element of pure counterfeiting has been introduced. Since the judges formulated the table before releasing the "Judicial Interpretation," adding the pure counterfeiting factor is deemed appropriate. Thirdly, whether the defendant’s infringement is a means of earning a livelihood reflects subjective malice, warranting a factor of 1.0 instead of 0.5. The judges did not provide a basis for assigning a factor to livelihood-based infringement.

Infringement as a means of profession is more malicious than measures taken to conceal and destroy evidence of infringement. Consequently, the factor is adjusted to 1.0. Fourthly, the judges deliberately distinguished between the country, province, and city. However, considering China as a unified market, there is no legal or factual basis for distinguishing between inter-city and inter-province scenarios. Given China’s vast territory, adopting a transnational, provincial, and municipal paradigm for determining standards may result in disparate judgments for the same case. Thus, the scope of infringement is deemed sufficient by encompassing domestic and cross-border sales. The factor for overseas sales is determined as 1.0, while the factor for domestic sales is set at 0.5, aligning with the principle of proportionality. Fifthly, the profit factor from infringement and "significant economic losses to the right holder" are recognized as two aspects of the same issue, leading to their unification into a single factor.

Additionally, in typical cases, the loss of goodwill suffered by the right holder due to infringement is considered a factor in punitive damages, prompting the addition of the loss of goodwill. Sixthly, due to a lack of cases in judicial practice considering the popularity of infringing products, the popularity factor of infringing products has been removed. The scope of the infringement factor is deemed adequate to reflect the influence of infringing products. Seventhly, destroying environmental resources and harming public interests has been consolidated into "socially serious circumstances."

At this juncture, deriving from the preceding analysis, we have identified nine factors that impact punitive damages, shown in **Table 1**. Within the table, the designations 1 to 9 signify distinct influencing factors. These factors have been distilled from existing judgments, drawing upon judicial interpretations. The indicator factors represent a nuanced delineation of different influencing factors contingent on varying degrees of "maliciousness" or "serious circumstances." The standard quantity denotes the multiplier for each factor under different levels of "maliciousness" or "serious circumstances." The standard quantity is predetermined based on the author’s trial experience and a comprehensive review of judgments, with coefficients calculated through a regression model formula. **Table 1** forms the cornerstone for the empirical analysis of diverse cases in the current research.

**Table 1:** Factors influencing punitive damages.

Factors	Number	Indicator Factors		Standard Quantity	Scope
**Maliciousness**	1	IP Recognition	Moderate influence at the provincial level	0.5	[0,1.5]
			Moderate influence nationwide	1.0	
			Moderate influence internationally	1.5	
	2	Plaintiff’s Awareness of Rights Holder’s IP	Maintains contractual relationships, such as licensing, distribution, agency, representation, or labor relations with the rights holder, and is aware of others’ intellectual property rights through negotiation.	0.5	[0,2]
			Fails to cease infringement after receiving a warning from the rights holder.	0.5	
			Continues infringing activities after receiving a court-issued injunction or following an infringement judgment in the first instance.	0.5	
			Engages in purely counterfeit activities (involving piracy, counterfeit registered trademarks) or applies for a similar trademark declared invalid.	0.5	
	3	Plaintiff’s Repeated Infringement	Infringes again after being punished by administrative authorities or being judicially determined to have infringed.	0.5	[0,1.5]
			Infringes again after being punished by administrative authorities or being judicially determined to have infringed, with a cumulative total of two prior instances.	1.0	
			Infringes again after being punished by administrative authorities or being judicially determined to have infringed, with a cumulative total of three prior instances.	1.5	
	4	Plaintiff Takes Measures to Conceal Infringement and Destroy Infringement Evidence		0.5	[0,0.5]
**Serious Circumstances**	5	Plaintiff Engages in Infringement as a Profession		1.0	[0,1]
	6	Duration of infringement	1–2 years	0.5	[0,1.5]
			2–3 years	1.0	
			3 years and above	1.5	
	7	Scope of infringement	Limited to domestic	0.5	[0,1]
			Extending to both domestic and international	1.0	
	8	Losses or profits from infringement	1–5 million	0.5	[0,1.5]
			5–10 million	1.0	
			Over 10 million	1.5	
	9	Consequences of infringement	Causes certain damage to the right holder’s reputation	0.5	[0,1.5]
			Endangers personal safety	0.5	
			Causes harm to public interests (such as damaging environmental resources)	0.5	

### 2.2 Establishment and modification of regression models

#### 2.2.1 Origins and selection of judicial documents

Our research employed a comprehensive data acquisition strategy. Initially, we used "IP" and "punitive damages" as search keywords on Pkulaw.com and wenshu.court.gov.cn. Additionally, interviews were conducted with judges from the Beijing IP Court, Shanghai Higher People’s Court, Hainan Higher People’s Court, Zhejiang Higher People’s Court, and Jiangsu Higher People’s Court. As of April 21, 2022, 3,478 judgments have been collected. Within these IP cases, only 79 judgments explicitly specified the application of punitive damages, and an additional 40 applied punitive damages without explicit specifications. A key challenge emerged throughout the screening and factor analysis process due to the lack of uniformity in judges’ adjudicative reasoning, which affected data extraction. To ensure the objectivity of the extraction standards, we implemented the following methodologies.

1. The statistical scope will include Judge opinions unequivocally endorsing punitive damages. Before the emergence of punitive damages, people’s courts sometimes utilized statutory damages. For example, specific courts initially supported the plaintiff’s claim for statutory damages while asserting that punitive damages should be judiciously awarded to curb infringement effectively. Subsequently, the relevant compensation basis was computed based on the goods’ unit price and sales quantity. Ultimately, the court adopted a comprehensive and discretionary approach to determine the compensation amount (Jiangsu Zhenjiang Intermediate People’s Court, 2020). In a judgment applying statutory damages, inclusion in the statistical scope occurs when the court examines the defendant’s subjective malice and the circumstances of the infringement. If the judgment explicitly articulates in the opinion section that punitive damages should be applied, it is included in the statistical analysis.
2. Judgments that do not explicitly invoke punitive damages in the judge’s opinion are excluded from the statistical scope. For instance, some courts contend that determining statutory damages hinges on the comprehensive assessment of factors such as the subject, the object, the infringing party, and the infringement behavior (Hangzhou Railway Transportation Court, 2019). Another example involves courts asserting that statutory and punitive damages should be concurrently applied. In such cases, the court might determine that the plaintiff’s actual losses and the defendant’s infringement profits are indeterminate, thus supporting the plaintiff’s claim for statutory damages. Additionally, the court might consider the defendant’s repeated infringements and decide to increase the amount of punitive damages (People’s Court of Haizhu District, 2018). These judgments explicitly characterize statutory damages as punitive, using the punitive nature as the rationale for judicial discretion. However, such judgments are excluded from the statistical analysis.
3. The identical facts described in diverse manners have been systematically categorized. Various judges articulate the same factual elements differently, necessitating a discerning analysis of the language used to ascertain their true import. First, consider the scope of infringement. Judges may not uniformly employ terms such as "overseas" or "domestic" to explicitly define the scope of infringement. Instead, they often use ambiguous expressions like "wide business scope" or "wide sales scale." In such cases, clarifying whether the infringement is domestic or foreign is crucial, and it is categorized accordingly as "domestic" or "overseas."
  Secondly, the duration of infringement is not consistently described. For instance, the Guangzhou IP Court employed the term "a lot of time" without specifying a specific duration, making it challenging to determine the Standard Quantity (Guangdong Intellectual Property Court, 2020). In another example, some courts briefly characterized the duration of infringement as "the defendant did not purchase the software involved or pay for software upgrades after 2006" (Shanghai Intellectual Property Court, 2017). Therefore, it is imperative to investigate when the infringement commenced to determine the facts of the case. Suppose no evidence is presented to demonstrate the cessation of the alleged infringement. In that case, the time calculation is based on the initiation of the first identified infringement until the conclusion of the judgment in the case.
  Lastly, the plaintiff’s awareness of the obligee’s IP varies. For instance, failure to cease infringement after legal proceedings is categorized as "failure to stop infringement after receiving a warning from the rights holder" (Shanghai Intellectual Property Court, 2017). Another scenario involves the plaintiff persisting in infringement after receiving a court order, classified as a "court injunction" rather than a "right holder warning" (Guangdong Provincial High People’s Court, 2019). Additionally, if the infringer’s behavior "may seriously damage the interests of various purchasers and growers and cause actual damage," it is categorized as "damaging the public interest" (Nanjing Intermediate People’s Court, 2020). The refinement of these categories enhances the precision of our statistical analysis.
4. Factors not explicitly addressed by the judge will be omitted from the count. Applying punitive damages involves many considerations, each case having unique infringement facts necessitating distinct assessments. When the judge does not explicitly outline the factors considered, the factors presented in the plaintiff’s and defendant’s arguments during prosecution and defense will not be considered. Instead, the judge’s rationale and factual determinations, specifically those articulated in the "this court holds" section, will be selectively extracted to capture the judge’s perspectives.
5. The actual compensation may surpass the plaintiff’s anticipated compensation, and the trial court might fully endorse the plaintiff’s expectations after calculating punitive damages based on the facts. For instance, the court determined that a trademark was declared invalid, and both parties were involved in trademark invalidation litigation. This situation encompassed "the infringer’s awareness of the existence of others’ IP after consultations between the two parties" and "continued infringement after the judgment of the first-instance court." Various scenarios with similar index factors need to be overlaid and computed in the calculation. The awarded compensation exceeds the plaintiff’s requested sum, thus garnering full support (Suzhou Intermediate People’s Court, 2020). Another example involves the court determining that the total compensation amount should be four times, with the lower limit of this range surpassing the plaintiff’s claimed amount by 30 million, leading to full support for the plaintiff’s claim (Zhejiang Higher People’s Court, 2021).
6. If the compensation basis is unspecified in the judgment, the compensation basis is initially examined, followed by statistical calculations. For instance, certain courts have decreed the application of punitive damages without specifying the multiplier for punitive damages. Instead, they directly provided the final compensation amount, including reasonable expenses and statutory damages (Jiangsu Higher People’s Court, 2019). Some courts asserted that the infringer engaged in repeated infringements, clearly stating that the law should apply punitive damages. However, due to the plaintiff’s failure to present evidence substantiating actual losses resulting from the infringement or the benefits acquired by the infringer, the court ultimately ruled that the defendant should compensate the plaintiff for economic losses based on comprehensive factors. This highlights the issue of exercising discretionary judgment under the guise of punitive damages. Certain courts determined that punitive damages should be applied in a case but did not clarify the compensation basis. Instead, they explicitly stated that the plaintiff’s claim fell within the scope of statutory damages and endorsed the claimed compensation (The Third People’s Court of Dongguan City, 2020). These types of judgments are excluded from the statistical analysis. Additionally, even in cases where the compensation basis is unspecified, punitive elements may still be present. For example, a court ruled that punitive damages should apply, awarding 80,000 yuan, including defense costs, without specifying the compensation basis (Zhenjiang Intermediate People’s Court, 2021). In such cases, this article initially assesses the judgment and then calculates various factors and multipliers for statistical purposes.
7. Valid agreements on punitive damages will be acknowledged, and statistics will be computed based on these agreements. In certain instances where there exists a pre-litigation mediation agreement or another pre-formulated agreement between the plaintiff and the defendant, assuming tort liability, the court has held that the amount agreed upon in the pre-agreement aligns with the principle of proportionality and maintains a balance of interests. As per the "Interpretation of the Supreme People’s Court on Several Issues Concerning the Application of Law in the Trial of Cases Involving Disputes over Infringement of Patent Rights (II)," when the right holder and the infringer have an agreement on the compensation or calculation method for patent infringement, and they claim in patent infringement litigation that the compensation should be determined based on the agreement, such claims should be supported (Shanghai Higher People’s Court, 2020).
8. Cases where punitive damages are arbitrarily determined will be included in the count. Specific scenarios are considered in batch litigation cases where defendants are financially vulnerable, the compensation basis is low, and the punitive damages are disproportionately high. For instance, during the fact-finding process, some courts regarded the plaintiff’s 48-yuan expenditure on purchasing infringing products for notarization as the infringer’s gains and directly imposed a penalty of five times the maximum punitive damages amount (Shanxi Higher People’s Court, 2020). Another example involves a court supporting the plaintiff’s demands based on the "Measures for Payment of Remuneration for the Use of Literary Works," applying a standard eight times the normal upper limit of royalties (300 yuan/thousand words) due to the defendant’s malice (Nanjing Intermediate People’s Court, 2019). Statistics for precedents in these situations are also collected to ensure alignment with the actual circumstances.

#### 2.2.2 Data sorting and analysis

**Fig 1:**
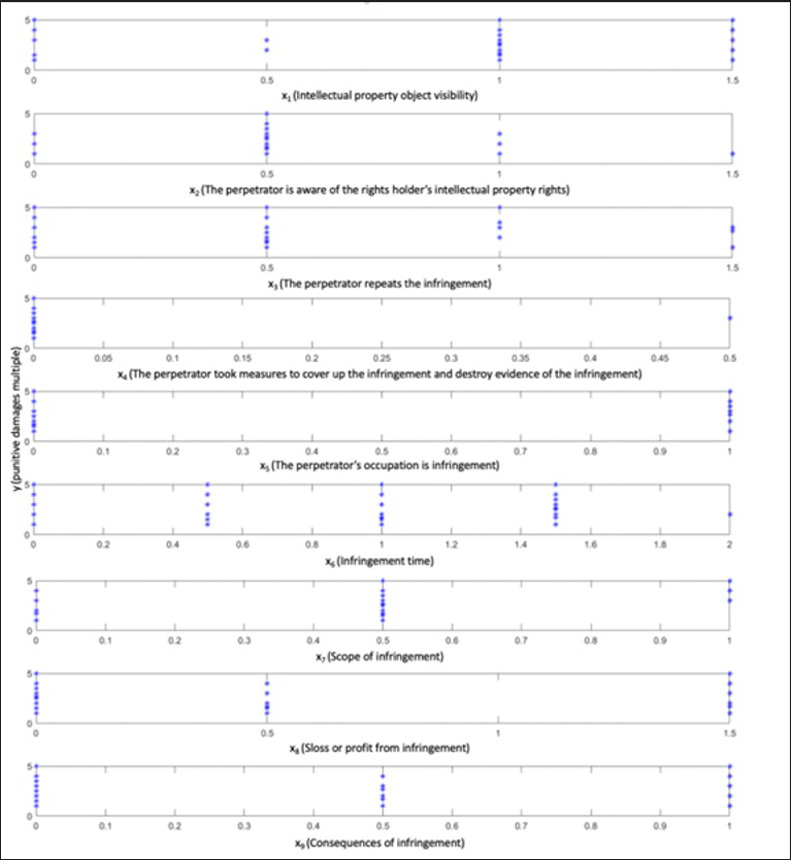
Scatter plot of punitive damages and various factors.

Utilizing the statistical method, we analyzed 79 relevant punitive judgments. More precisely, the study involved tabulating the influencing factors of punitive damages and the actual multiplier in the judgment. Subsequently, we constructed a scatter plot illustrating the relationship between punitive damages and each factor. The numerical values representing the impact factor of the punitive damage multiplier in each judgment, and the corresponding actual judgment values are documented in **Table 2.** A scatter plot depicting judgment compensation multiplier, and the nine primary factors is presented for the existing 79 datasets, as illustrated in **Fig 1.**

**Table 2:** Valuation of influencing factors and actual judgment values in 79 punitive damages judgments.

Number	Area	Document number	IP Recognition	Plaintiff’s Awareness of Rights Holder’s IP	Plaintiff’s Repeated Infringement	Plaintiff Takes Measures to Conceal Infringement and Destroy Infringement Evidence	Plaintiff Engages in Infringement as a Profession	Duration of infringement	Scope of infringement	Losses or profits from infringement	Consequences of infringement	Actual judgment value
1	Beijing	(2019) SPC IP Civil Final No.562	0	0.5	1	0	1	1.5	1	1.5	0	5
2	Beijing	(2015) Jing IP Court Civil First Instance No.1677	1	0.5	0	0	0	0	0	0	0	2
3	Beijing	(2018) Jing IP Court Civil Appeal No.4666	1.5	0.5	0	0	0	0.5	0.5	0.5	0.5	3
4	Beijing	(2020) SPC IP Civil Final No.765	1	0.5	0.5	0	1	1	0	0	0.5	2
5	Beijing	(2017) Jing IP Court 0107 Civil Final No.18968	1.5	0	0	0	1	1.5	0.5	0.5	1	1
6	Beijing	(2018) Jing IP Court 73 Civil Final No.2132	1	0.5	0	0	0	0.5	0.5	0.5	0	1.5
7	Beijing	(2021) SPC IP Civil Final No.816	1	0.5	0	0	1	1	0	0.5	0.5	3
8	Guangdong	(2019) Yue Civil Appeal No.147	1	0.5	0	0	1	1.5	1	1.5	0.5	3
9	Guangdong	(2020) Yue 73 Civil Final No. 2442	1	1	0.5	0	0	2	0.5	0	0.5	2
10	Guangdong	(2019) Yue Civil Final No.477	1	1	0	0.5	1.0	0.5	0.5	1.5	0.5	3
11	Guangdong	(2020) Yue IP Court Civil 73 First Instance No.57	0	0	0.5	0	0	1.5	0.5	0	0	3.0
12	Guangdong	(2020) Yue 06 Civil Final No.1034	1	0.5	0.5	0	0	1.0	0	0	1	2.0
13	Guangdong	(2018) Yue 73 Civil Final No.4425	1	0.5	0.5	0	0	1.5	0	0	0.5	1.0
14	Guangdong	(2020) Yue 1973 Civil First Instance No.4738	1	0.5	0.5	0	0	1.5	0.5	0	0.5	2.0
15	Guangdong	(2020) Yue 1973 Civil First Instance No.4735	1	0.5	0.5	0	0	1.5	0.5	0	0.5	2.0
16	Guangdong	(2020) Yue 1973 Civil First Instance No.4731	1.5	0.5	0.5	0	1	1.5	0.5	0	0	2.0
17	Guangdong	(2020) Yue 0604 Civil First Instance No.9522	1	0.5	0.5	0	0	1.5	0.5	0	0	2.5
18	Guangdong	(2020) Yue 1973 Civil First Instance No.4736	1.5	0.5	0.5	0	0	1.5	0.5	0	0.5	2.0
19	Guangdong	(2020) Yue 03 Civil Final No.16190	1	0.5	0	0	0	0	0.5	0	0.5	3.0
20	Guangdong	(2020) Yue 01044 Civil First Instance No.6217	1.5	0.5	0	0	0	0	0.5	0	0	4.0
21	Guangdong	(2020) Yue 0111 Civil First Instance No.5194	0.5	0.5	0.5	0	0	0.5	0.5	0	0.5	2.0
22	Guangdong	(2017) Yue 73 Civil First Instance No.2239	1	0.5	0	0	1	1.5	0.5	1.5	1	3.0
23	Guangdong	(2020) Yue 0105 Civil First Instance No.12586	1	0.5	0	0	1	0.5	0.5	0	0	4
24	Guizhou	(2021) Qian Civil Final No.1180	1	0.5	0	0	0	1	0.5	0	1	5.0
25	Guizhou	(2021) Qian 03 Civil First Instance No.25	1	0.5	0	0	0	0.5	0.5	0	0.5	3.0
26	Chongqing	(2019) Yu 05 Civil First Instance No.1225	0.5	0.5	0	0	0	0.5	0.5	0	0.5	3.0
27	Henan	(2021) Yu IP Court Civil Final No.69	0.5	0.5	1	0	0	1.5	0.5	0	0.5	3.0
28	Henan	(2019) Yu 11 IP Court Civil First Instance No.7	0.5	0.5	0	0	0	1.5	0.5	0	0.5	2
29	Henan	(2020) Yu 01 IP Court Civil First Instance No.99	1	0.5	0	0	1.0	0.5	0	0	0.5	1.0
30	Henan	(2020) Yu 17 IP Court Civil First Instance No.263	1	0.0	0	0	0	1	0	0	0.5	2
31	Hubei	(2021) Yue IP Court Civil Final No.597	1	0.5	0.5	0	0	1	0.5	0	0	1.5
32	Hubei	(2021) Yue 06 IP Court Civil First Instance No.77	0	0.5	0.5	0	0	1	0.5	0	0	1.5
33	Hunan	(2020) Xiang 01Civil Final No.1736	1	0.5	0	0	0	0.5	0.5	0	1	2
34	Jiangsu	(2019) Su Civil Final No.1316	1	0.5	0.5	0	1	1	0	1.5	0.5	3
35	Jiangsu	(2020) Su Civil First Instance No.560	1	1.5	0	0	0	1.5	0	1.5	0	1
36	Jiangsu	(2020) Su 01 Civil First Instance No.226	0	0.5	0	0.5	0	1.5	0.5	0.5	0.5	3
37	Jiangsu	(2019) Su Civil Final No.95	1	0.5	0	0	0	0	0.5	1.5	0.5	2
38	Jiangsu	(2020) Su 13 Civil First Instance No.484	1	0.5	1.5	0	1	1.5	0.5	0	0.5	2.7
39	Jiangsu	(2018) Su 05 Civil First Instance No.115	1	0.5	0.5	0	0	1	0.5	1.5	0.5	1.7
40	Jiangsu	(2018) Su 08 Civil First Instance No.266	1	0.5	0.0	0	1	1.0	0.5	0	0.5	2.0
41	Jiangsu	(2020) Su 04 Civil First Instance No.344	1.5	0.5	0.0	0	1	1	0.5	0	0	2.0
42	Jiangsu	(2020) Su 5 Civil First Instance No.271	1.5	1.0	0	0	0	1.5	0.5	1.5	0.5	1.0
43	Jiangsu	(2019) Su Civil Final No.476	1	0.5	0.5	0	0	1.5	0	0.5	0.5	1.7
44	Jiangsu	(2019) Su 05 IP Court First Instance No.250	1.5	0.5	0.5	0	1	1.5	0.5	0.5	0.5	3.0
45	Jiangsu	(2020) Su IP Court Final No.60	1.5	0.5	0	0	0	0.5	0.5	1.5	0	2.0
46	Jiangsu	(2021) Su 13 Civil Final No.2400	0	0.5	0.5	0	0	1	0.5	0	0	1.5
47	Jiangsu	(2019) Su 05 IP Court First Instance No.643	1	0.5	0	0	0	1.5	0.5	1.5	0.5	2.0
48	Liaoning	(2021) Liao Civil Final No.230	1	0.5	0	0	0	1.5	0.5	0	0	1.0
49	Shandong	(2021) Lu 1402 Civil First Instance No.4758	0	0.5	0.5	0	0	1	0.5	0.0	0.0	1
50	Shandong	(2021) Lu 15 Civil First Instance No. 216	0	0.5	0.0	0	0	1	0.5	0.0	0.0	1.5
51	Shandong	(2020) Lu 07 Civil First Instance No. 1261	1	0.5	0	0	0	0	0.0	0	0	2.0
52	Shanxi	(2020) Jin 04 Civil First Instance No. 78	1.5	0.5	0	0	0	0	0.5	0	0	5.0
53	Shanxi	(2020) Jin 04 Civil First Instance No. 71	1.5	0.5	0	0	0	0	0.5	0	0	5.0
54	Shanxi	(2020) Jin 4 Civil First Instance No. 60	1.5	0.5	0	0	0	0	0.5	0	0	5.0
55	Shanxi	(2020) Jin Civil Final No.13	1	0.5	0	0	0	0.0	0.5	0	1.0	5.0
56	Shaanxi	(2021) Shan 01 IP Court Civil First Instance No.1168	1	0.5	0.5	0	0	1.5	0.5	0	0	3.0
57	Shanghai	(2018)Hu 115 Civil First Instance No.53351	1	0.5	0	0	1.0	0.5	0.5	0.5	0.0	3
58	Shanghai	(2017) Hu 73 Civil First Instance No. 712	0	0.5	0	0	1.0	1.5	0.5	1.5	0	5.0
59	Shanghai	(2021) Hu 73 Civil Final No. 611	1	0.5	0.5	0	0	0.5	0.5	0.5	0.5	2
60	Sichuan	(2021) Chuan 0193 Civil First Instance No. 5246	1.5	1	1.5	0	1.0	1.5	0.5	0.5	0	3.0
61	Sichuan	(2019) Chuan 01 Civil First Instance No. 2428	1	0.5	1.5	0	0	1.5	0.0	0	1.0	1.0
62	Yunnan	(2021) Yun 29 Civil First Instance No. 891	0	0.5	0.5	0	0.0	1.0	0.5	0.0	0.0	1
63	Yunnan	(2021) Yun 29 Civil First Instance No.922	0	0.5	0.5	0	0	0	0.5	0	0	1
64	Yunnan	(2021) Yun 29 Civil First Instance No.817	0	0.5	0.5	0	0.0	0.5	0.5	0.0	0.0	1
65	Yunnan	(2021) Yun 29 Civil First Instance No.827	0	0.5	0.5	0	0.0	0.5	0.5	0.0	0.0	1
66	Yunnan	(2021) Yun 29 Civil First Instance No.652	0	0.5	0.5	0	0	0.5	0.5	0.0	0.0	1
67	Yunnan	(2021) Yun 29 Civil First Instance No.680	0	0.5	0.5	0	0	0.5	0.5	0.0	0.0	1
68	Yunnan	(2021) Yun 25Civil First Instance1220	1	0	0.5	0	1	0	0.5	0	0	1.0
69	Zhejiang	(2020) Zhe 01Civil Final No.5872	1	0.5	1	0	1	0.5	0	0.5	0.5	2.0
70	Zhejiang	(2021) Zhe 02 Civil First Instance1No.093	0	0.5	0.0	0	0	1.0	1.0	0.5	0	4.0
71	Zhejiang	(2021) Zhe 0212 Civil First Instance No.15284	1	0.5	0.5	0	0	0.5	0.5	0	0	5
72	Zhejiang	(2021) Zhe Civil Final No.294	1.5	0.5	0.5	0	1	1.5	1	1.5	1.0	4.0
73	Zhejiang	(2019) Zhe 07 Civil Final No.721	1	0.5	0.5	0	1	1.5	0.5	0	0.5	3.0
74	Zhejiang	(2020) Zhe 08 Civil First Instance No.108	1	0.5	1	0	1	1.5	0.5	0	0	3.5
75	Zhejiang	(2019) Zhe 01 Civil First Instance No.412	1.5	0.5	0.5	0	1	1.5	0	1.5	0.5	4.0
76	Zhejiang	(2019) Zhe 01Civil Final No.4919	1	0.5	0.5	0	0	1.5	0.5	0	0.5	2.0
77	Zhejiang	(2021) Zhe 01 Civil Final No.1364	0.5	0.5	0.5	0	0	1.5	0.5	0	0.5	3.0
78	Zhejiang	(2019) Zhe 8601 Civil First Instance No.1611	1	0.5	0	0	0	0.5	0.5	0.5	0	1.5
79	Zhejiang	(2020) Zhe 03 Civil Final No.161	1.5	0.5	1.5	0	0.0	1.5	0.5	0.0	0.0	3

Based on the information presented in **Table 2,** we create a scatter plot depicting punitive damages concerning various factors. In regression analysis, the primary purpose of a scatter plot is to preliminarily identify potential correlations between variables and establish a foundation for subsequent regression analysis. Suppose the scatter plot exhibits a discernible trend or pattern among the data points. In that case, it suggests a potential correlation between the variables, prompting further investigation through regression analysis to ascertain the specific nature of the relationship. This analysis allows the selection of an appropriate function to model and fit the data points.

#### 2.2.3 Establishment of the equation

**Fig 1**, shows an evident linear relationship in the scatter plots of the nine major factors across the 79-judgment sets and the multiplier in judgments. Typically, a significance level of 0.05 is employed, leading to the derivation of the multiple linear regression equation based on these 79 punitive damages judgments:

$$min\sum_{j=1}^{m}{\left(y_{j}-\sum_{i=1}^{n}a_{i}x_{ij}-a_{i}x_{ij}-\varepsilon \right)}^{2}$$

The equation calculates the squared difference between the predicted and actual values for each data point, sums these squared differences, and identifies the parameter values that minimize this sum. As the squared difference is inherently positive, minimizing the sum indicates that the difference between the predictions and the actual result of this formula is minimized. Examining the equation reveals a positive relationship between punitive damages and factors such as the reputation of IP assets, the plaintiff’s efforts to conceal infringing behavior and destroy evidence, the plaintiff engaging in infringement as a profession, the scope of infringement, and the consequences of infringement, including losses or profits.

While the equation serves as an objective description, it does not accurately reflect the dynamics of judicial application of punitive damages. In other words, the regression equation, without constraints, fails to fit the factual scenario in calculating punitive damages. Consequently, the statistical regression equation derived from these 79 judgments does not apply to real-world scenarios. Therefore, additional conditions need to be introduced, specifically ensuring that the weighting coefficient of each factor remains positive. This transforms the problem from a statistical issue into a mathematical optimization problem.

Let y be an observable dependent variable in actual decision-making, influenced by n (*n* ϵ *N*_+_) independent variables X_1_, X_2_, …, X_n_, and an error term *ε*. Assume that Y exhibits the following weighted linear relationship with x_1_, x_2_, …, x_n_:

$$Y=a_{1}X_{1}+a_{2}X_{2}+\cdots +a_{n}X_{n}+\varepsilon .$$

Consider groups of observed values (x_11_, x_21_, …x_n1_, y_1_,), (x_12_, x_22_, …x_n2_, y_2_,), …(x_1m_, x_2m_, …x_nm_, y_m_,), that should adhere to the following relationship:

$$\left\{\begin{array}{c} y_{1}=a_{1}x_{11}+a_{2}x_{21}+\cdots +a_{n}x_{n1}+\varepsilon ,\\ y_{1}=a_{1}x_{12}+a_{2}x_{22}+\cdots +a_{n}x_{n2}+\varepsilon ,\\ \vdots \\ y_{m}=a_{1}x_{1m}+a_{2}x_{2m}+\cdots +a_{n}x_{nm}+\varepsilon . \end{array}\right.$$

The standard for the punitive damage multiplier comprises nine factors, as indicated in **Table 1**. There are 79 judgments, as presented in **Table 2.** From **Table 2**, we extract the data for the independent variable factors and the dependent variable (i.e., multiplier). Based on this, we can formulate the following constrained least squares optimization equation, where the lower bound for each of the nine factors is set at 0.1:

$$min\sum_{j=1}^{m^{\prime }}{\left(y_{j}-\sum_{i=1}^{9}a_{i}x_{ij}-\varepsilon \right)}^{2}$$

$$s.t.\;a_{i}\geq 0.1,\;i=1,2,\;\ldots ,9$$

Utilizing R software version 4.2.2 and the ’lm’ function to solve this equation, we obtained an adjusted R-squared value of 0.1104. Subsequently, by adjusting the intercept to minimize the discrepancy, the resulting values are as follows.

*a*_1_ *= 0*.*5735*, *a*_2_ *= 0*.*1*, *a*_3_ *= 0*.*1*, *a*_4_ *= 1*.*0946*, *a*_5_ *= 0*.*4162*, *a*_6_ *= 0*.*1*, *a*_7_ *= 1*.*8205*, *a*_8_ *= 0*.*0571*, *a*_9_ *= 0*.*1296*, *ε = 0*.*7879*.

Applying the revised 79 samples to this equation, we can get the new punitive damages equation as follows:

Y = 0.5735×Reputation of Intellectual Property Assets + 0.1×The plaintiff is aware of the obligee’s IP + 0.1×The plaintiff repeats the infringement + 1.0946×The plaintiff takes measures to conceal infringing behavior and destroy evidence of infringement + 0.4162×The plaintiff engaging in infringement as a profession + 0.1 ×Duration of infringement + 1.8205 ×Scope of infringement + 0.0571×Losses or profit from infringement + 0.1296×Consequences of infringement + 0.7879.

**Fig 2:**
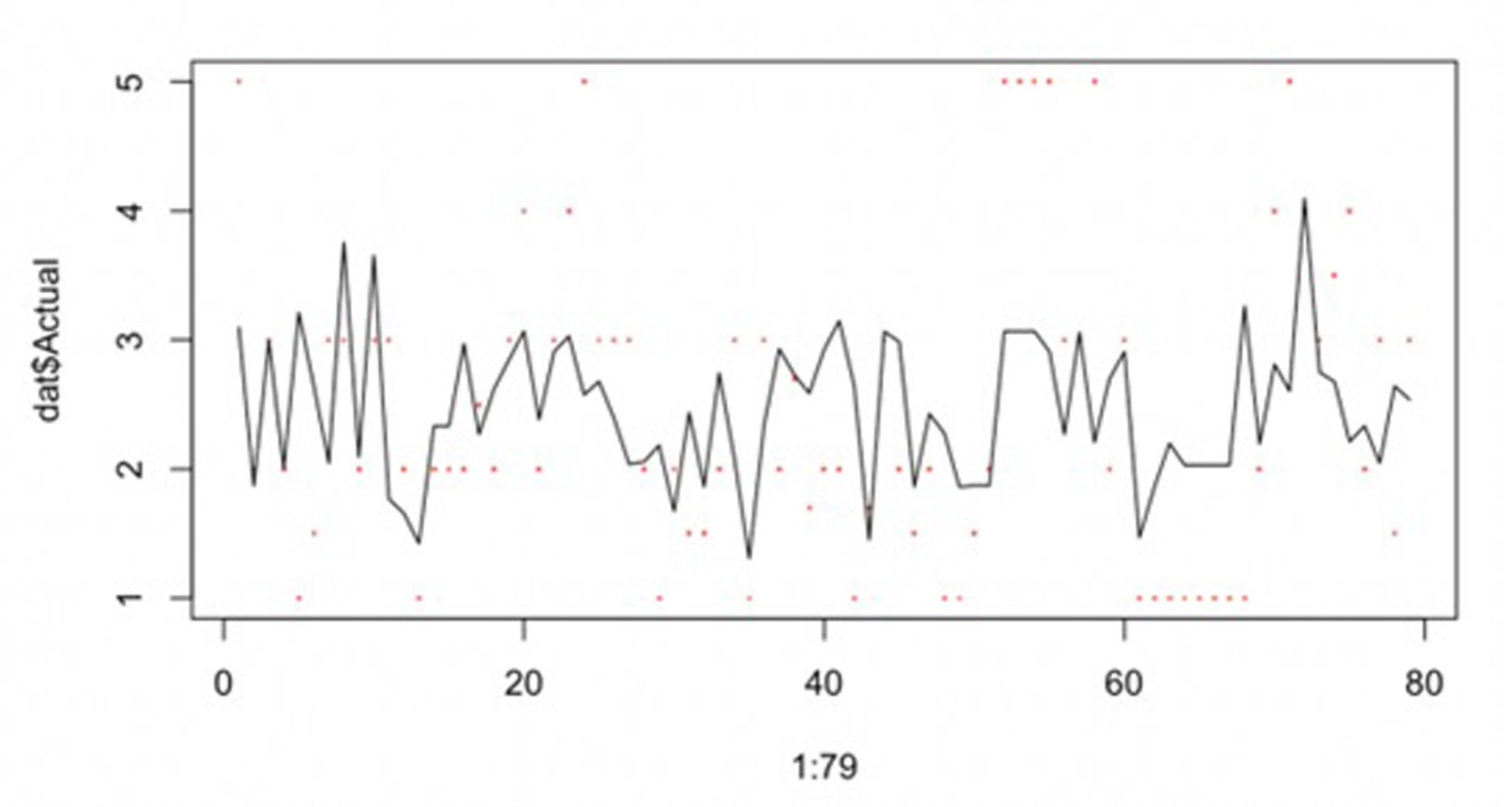
Multipliers of least square regression and true output multipliers.

Using the equation, we produced the multiplier from the least square regression and the actual output multiplier, as shown in **Fig 2**. The red star points represent the actual multipliers for 79 cases, while the black line represents the multiplier fitted.

#### 2.2.4 Results discussion of least squares model

This article has meticulously selected 79 judgments deemed applicable to punitive damages from 3478 IP judgments. Through quantitative analysis, it aims to elucidate the nine factors that constitute "maliciousness" and "serious circumstances." Specifically, this model reveals varying weights for different influencing factors.

Firstly, the "scope of infringement" coefficient remains the largest at 1.8205, underscoring its consistent prominence in determining the punitive damage multiplier. The penalty multiplier of 0.9103 (resulting from the product of the factor’s base value of 0.5 and the weight of 1.8205) can be considered the fundamental penalty multiplier when infringement is confined to the country. A multiplier of 1.8205 (product of the factor’s base value of 1 and the weight of 1.8205) is obtained for domestic and international occurrences.

Secondly, the coefficient of "the plaintiff taking measures to conceal infringing behavior and destroy evidence of infringement" is 1.0946, maintaining its significance compared to Eq (2). Whenever the infringer undertakes measures to conceal infringement and destroy evidence, a penalty multiplier of 0.5473 (resulting from the product of the basic factor value of 0.5 and the weight of 1.0946) is assigned. The proportions of this factor and "scope of infringement" exceed 1.

Thirdly, the "reputation of Intellectual Property Assets" proportion is 0.5735, retaining its original importance ranking. When IP assets wield a particular influence within the province, a penalty multiplier of 0.2868 (resulting from the product of the factor’s base value of 0.5 and the weight of 0.5735) is obtained. When IP assets hold influence overseas, a penalty multiplier of 0.5735 (factor base value of 1 and weight of 0.5735) is secured. Ranked fourth is "the plaintiff engaging in infringement as a profession," where a penalty of 0.4162 is incurred (product of the factor’s base value of 1 and the weight of 0.4162).

One of the main drawbacks of the correction above method is that it sometimes leads to nonsensical predictions due to the need to consider constrained models to avoid obtaining potentially negative prediction values. Nevertheless, structurally removing negative correlations or forcing them to be non-negative is unreasonable from a statistical perspective. Statistical analysis must respect the data and its theoretical structure. Ordinary linear modeling methods yield potentially negative predictions because the linear model assumes the conditional response variable is normally distributed. A normal distribution can take any real value. This is precisely why linear regression is unsuitable for modeling binary or categorical response outcomes, and it can be reasonably argued that it is also inapplicable here. The response variable is structurally bounded below by zero.

## 3. Enhancement of the regression model

In order to solve the drawbacks, we consider using a generalized linear model (GLM) instead of a conventional linear model, for which we opt to use the beta regression model [21]. However, since beta random variables must lie within the interval [0,1], we must transform the response variable to fall within this range. This is merely a simple scaling because we consider the structural upper limit to be 12 and know that the structural lower limit is already 0. By utilizing R software version 4.2.2 and employing the "betareg" function to solve this equation, where two additional interaction terms (IP*Repeat, IP*Duration) have been included in the revised model, we obtained a pseudo-R-squared value of 0.5076, which demonstrates a significant improvement over the previous linear model(adjusted R-squared value: 0.1104). The new punitive damages equation is as follows:

$$Y=\frac{e^{\mu }}{1+e^{\mu }},$$

where *μ = logit(Y)* = 1.4285×Reputation of Intellectual Property Assets + 0.3961×The plaintiff is aware of the obligee’s IP ‐ 0.5680×The plaintiff repeats the infringement ‐ 0.6479×The plaintiff takes measures to conceal infringing behavior and destroy evidence of infringement + 0.2899×The plaintiff engaging in infringement as a profession + 1.2677×Duration of infringement + 1.0052×Scope of infringement ‐ 0.0415×Losses or profit from infringement + 0.3419×Consequences of infringement + 0.7382×Reputation of Intellectual Property Assets×The plaintiff repeats the infringement ‐ 1.5460×Reputation of Intellectual Property Assets×Duration of infringement ‐ 3.4102.

**Fig 3:**
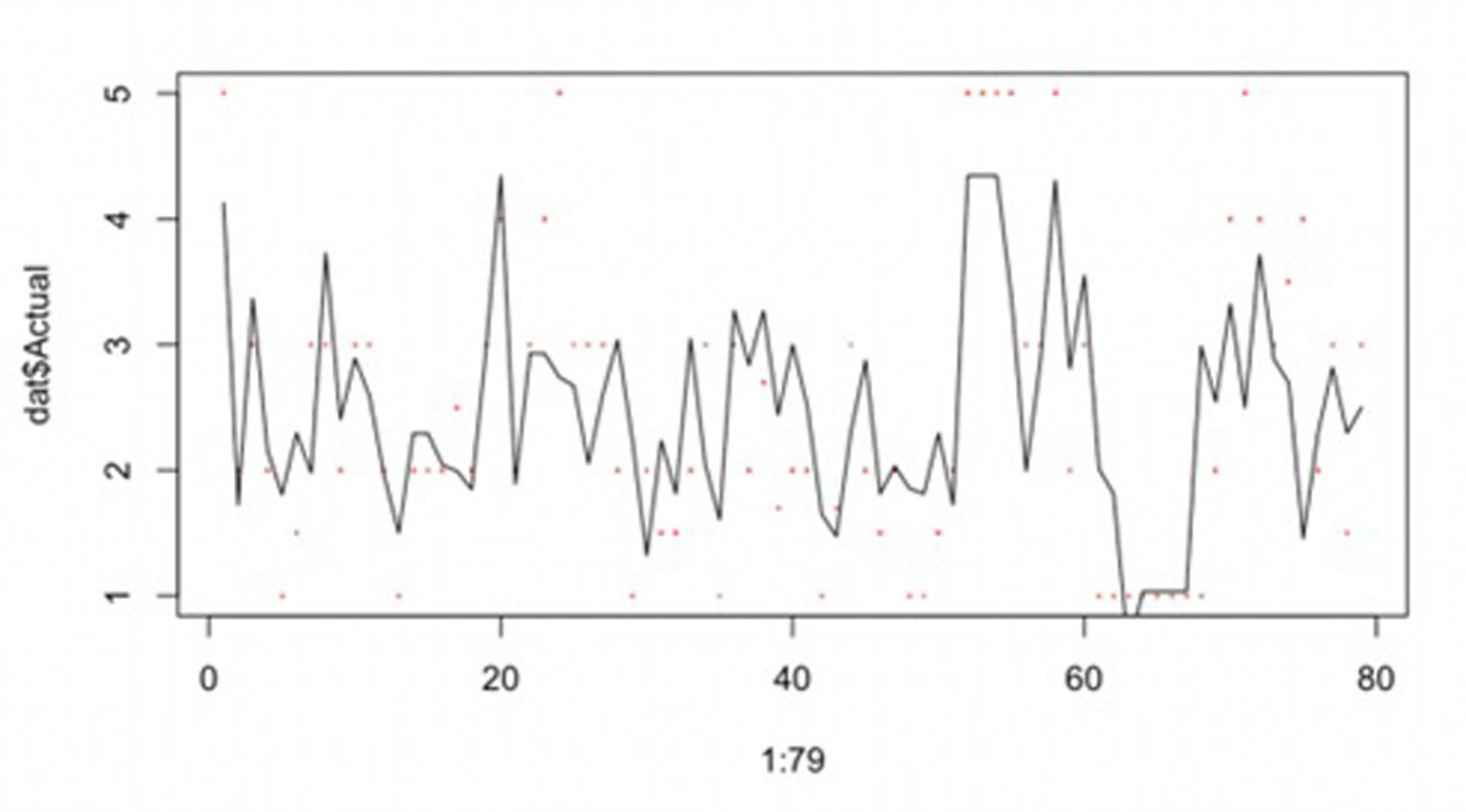
Multipliers of beta GLM and true output multipliers.

Since our negative coefficients are on top of the exponents in the equation, the effect of each factor on the punitive damages judgment multiplier is positively affected after the calculation. By the punitive damages equation, we get **Fig 3.**

We can see that the agreement between the actual data and the red dots in **Fig 3** will be higher compared to **Fig 2**, implying that the data model may be more accurate or the actual situation is close to the prediction.

**Fig 4:**
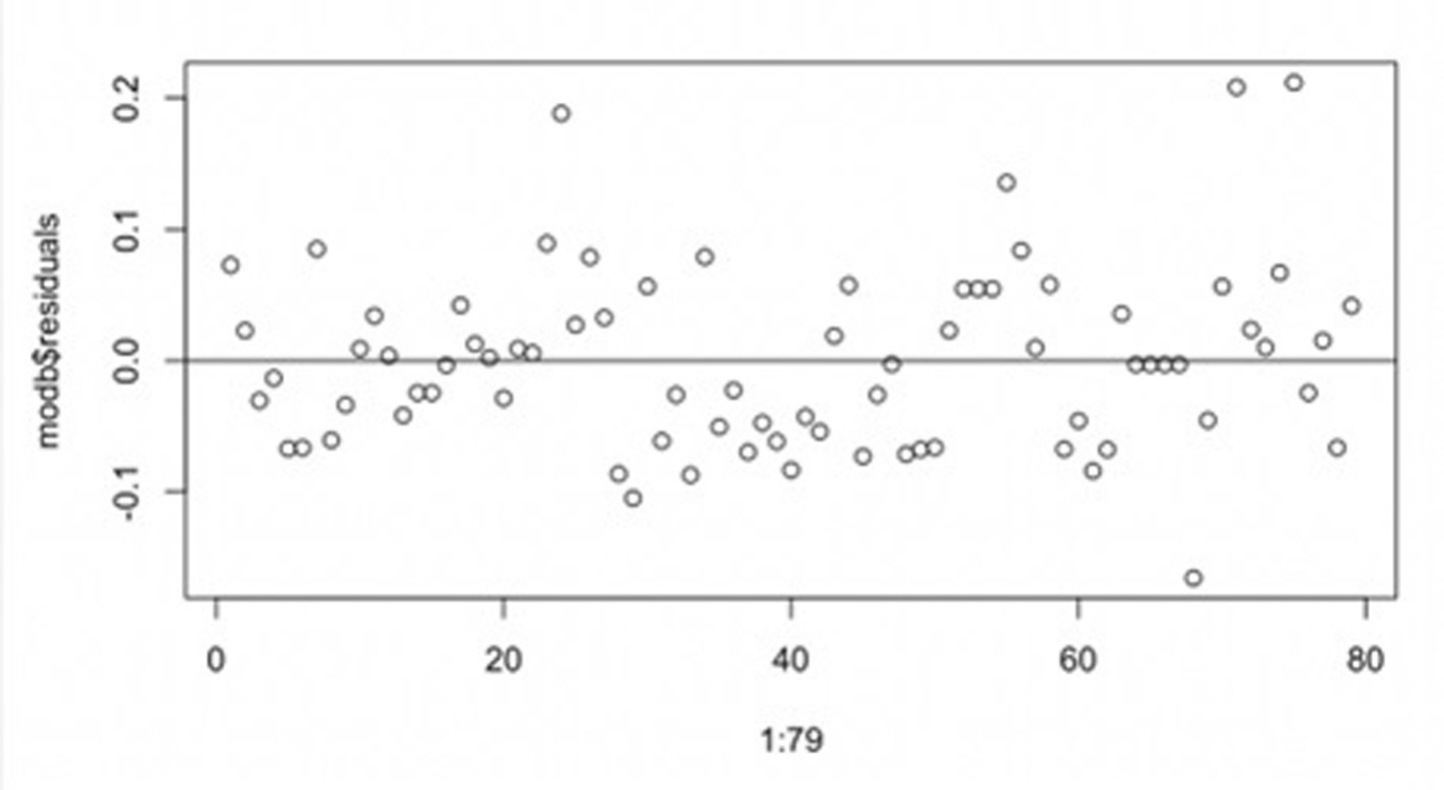
Residuals of beta GLM.

In addition, residual plots are also crucial tools in statistical modeling for assessing the fit of a model. By generating **Fig 4**, we can verify the validity of model assumptions and identify potential outliers or patterns.

We generated **Fig 4** and see that the residuals are evenly distributed on both sides of the horizontal axis, suggesting that the model does not exhibit any apparent systematic bias. This implies that the mean of the residuals is close to zero, which aligns with the regression model’s assumptions. Most residual values are concentrated between -0.1 and 0.1, indicating that the discrepancies between the predicted and actual values are relatively small. Furthermore, the absence of any discernible curvilinear trend in the residuals suggests that the relationship between the data and the predictor variables is linear, thereby supporting the application of the Beta regression model. Besides, there are no apparent anomalous residual values in **Fig 4**, meaning all data points are relatively evenly distributed within the expected error range. This reduces the likelihood of overfitting or underfitting and shows that the model has good generalization to the data.

### 3.1 Results discussion of Beta GLM

Utilizing the Beta Generalized Linear Model, we see that among the nine factors influencing punitive damages, the key factors are the Reputation of Intellectual Property Assets, the Duration of infringement, and the scope of infringement.

Firstly, the "scope of infringement" coefficient remains at 1.0052, underscoring its consistent prominence in determining the punitive damage multiplier. The penalty multiplier of 0.623 (resulting from $\text{Y}=\frac{{\text{e}}^{\mu }}{{1+\text{e}}^{\mu }}$ where μ is the product of the factor’s base value of 0.5 and the weight of 1.0052) can be considered the fundamental penalty multiplier when infringement is confined to the country. A multiplier of 0.732 (where μ is the product of the factor’s base value of 1 and the weight of 1.0052) is obtained for domestic and international occurrences.

Secondly, although the coefficients for Reputation of Intellectual Property Assets (1.4285) and Duration of infringement (1.2677) are both significant, the interaction coefficient between Reputation of Intellectual Property Assets and Duration of infringement (-1.5460) is also substantial. Therefore, we believe that the effects of the Reputation of Intellectual Property Assets and the Duration of infringement cannot be distinguished. However, following mathematical recalculations, we can conclude that with higher values of duration of infringement, there appears to be an inference that the increased reputation of intellectual property assets correlates with smaller judgment amounts. Similarly, with elevated Reputation of Intellectual Property Assets values, it can be inferred that more significant Duration of infringement values are linked to reduced judgment amounts.

Despite the technical and applicational differences between the two models, there is a noticeable overlap in identifying key variables, particularly in the scope of infringement and the reputation of intellectual property assets. The extensive scope of infringement typically indicates a broader market impact and more severe economic losses. This factor has been emphasized in both models, suggesting that irrespective of the statistical method employed, courts are likely to impose heavier economic penalties for widespread infringement activities. Simultaneously, the reputation of intellectual property assets is considered a significant factor in both models, reflecting the market value of IP and the potential losses incurred when infringed.

This consistency suggests that the legal framework’s emphasis on certain factors is consistent regardless of the statistical analysis method used. This aids in understanding the general application and enforcement of legal rules. Also, by recognizing these key variables, legal professionals can better formulate strategies for their clients, especially when preparing litigation materials and arguments. Emphasizing these key factors can enhance the persuasiveness of a case.

### 3.3 Verification of unspecified cases

Combining the 40 cases in which punitive damages are applicable but no specific punitive damages judgment is provided, the predicted judgment values are derived by applying Eq (4) from this article (refer to No. 1 to No. 40 in **Table 3**). Additionally, based on the circumstances of five typical cases, instructions for judgment are provided (refer to No. 41 to No. 45 in **Table 3**). These 45 cases collectively verify the efficacy of the punitive damages equation presented in this article, with **Table 3** detailing the scores assigned to each factor and the corresponding predicted judgment values.

**Table 3:** Estimates of verdicts in punitive damage cases.

Number	Area	Document number	IP Recognition	Plaintiff’s Awareness of Rights Holder’s IP	Plaintiff’s Repeated Infringement	Plaintiff Takes Measures to Conceal Infringement and Destroy Infringement Evidence	Plaintiff Engages in Infringement as a Profession	Duration of infringement	Scope of infringement	Losses or profits from infringement	Consequences of infringement	Predict judgment value
1	Anhui	(2021) Wan 04 Civil First Instance No.464	1	0.5	0.5	0	0	0.5	0.5	0	0	2.3
2	Anhui	(2020) Wan 0291 Civil First Instance No.466	0.5	0.5	0.5	0	0	1.5	0	0	0.5	1.6
3	Fujian	(2018) Min 0582 Civil First Instance No.14301	1	0.5	0	0	0	0	0.5	0	0	2.4
4	Gansu	(2019) Gan Civil Final No.269	1	0.5	0	0	1	1.5	0	0.5	1	2.2
5	Guangdong	(2019) Yue 03 Civil Final No.25451	1	0.5	0	0	0	1.5	0.5	0	0	2.3
6	Guangdong	(2021) Yue 20 Civil Final No.683	1	0.5	0	0	0	0	0	0.5	0.5	2.4
7	Guangdong	(2018) Yue 0306 Civil First Instance No.19497	1	0.5	0	0	0	1.5	0.5	0	0.5	1.7
8	Guangdong	(2020) Yue Civil Final No.1034	1	0	0.5	0	0	0.5	0.5	0	0	2.4
9	Guangdong	(2018) Yue 0105 Civil First Instance No.19324	0	0	1	0	0	1.5	0	0	0	2.3
10	Guangdong	(2021) Yue Civil Final No.679	1	0.5	0.5	0	0	1	0.5	0	0	1.3
11	Guangdong	(2019) Yue 0192 Civil First Instance No.24305	0	0.5	0	0	0	1	0.5	0	0	2.4
12	Guangzhou	(2021) Yue 73 Civil Final No.5842	1	0	0	0	0	0	0.5	0	0	1.8
13	Guangzhou	(2021) Yue 73 Civil Final No.5843	1	0	0	0	0	0	0.5	0	0	2.2
14	Guangzhou	(2021) Yue 73 Civil Final No.5844	1	0	0	0	0	0	0.5	0	0	2.2
15	Guangzhou	(2021) Yue 73 Civil Final No.5845	1	0	0	0	0	0	0.5	0	0	2.2
16	Henan	(2020) Yu IP Court Civil Final No.230	1	0.5	1.5	0	0	1.5	0.5	0	0.5	2.2
17	Jiangsu	(2021) Su 01 Civil First Instance No.850	0.5	0	0	0	0	0	0.5	0.5	1	2.6
18	Jiangsu	(2019) Su 11 Civil First Instance No.155	0.5	0	0	0.5	0	0	0.5	0	0.5	2.1
19	Jiangsu	(2020) Su 04 Civil First Instance No.153	0.5	0.5	0	0	0	1.5	0.5	0	0	2.5
20	Jiangsu	(2020) Su 11 Civil First Instance No.186	0.5	0	0	0	0	0	0.5	0	0.5	2.1
21	Jiangsu	(2020) Su 11 Civil First Instance No.64	1	0.5	0	0	0	0	0.5	0	0.5	2.0
22	Jiangsu	(2021) Su 11 Civil First Instance No.78	1	0.5	0	0	1	0	0	0	0	2.3
23	Jiangsu	(2020) Su 13 Civil First Instance No.484	1	1	1.5	0	0	0	0	0	0.5	2.0
24	Jiangsu	(2019) Su 01 Civil First Instance No.2672	1	0.5	0	0	0	0	0.5	0	0.5	1.8
25	Jiangsu	(2018) Su 05 Civil First Instance No.572	1	0.5	0.5	0	1	1.5	0.5	0	0	2.3
26	Jiangsu	(2021) Su 11 Civil First Instance No.78	1	0.5	0	0	0	0	0.5	0	0	2.9
27	Liaoning	Liao 0192 Civil First Instance No.2567	1	0	0	1	0.5	0.5	0.5	0	0	2.2
28	Shandong	(2021) Lu Civil Final No.1307	1.5	0.5	1.5	0	0	1.5	0	1.5	0.5	3.5
29	Shanxi	(2019) Shan 07 IP Court Civil First Instance No.33	1	0.5	0.5	0	0	1.5	0.5	0	1	2.3
30	Shanghai	(2020) Hu Civil Final No.555	1	0.5	1	0	0	1.5	0.5	0.5	0	2.5
31	Shanghai	(2020) Hu 0104 Civil First Instance No.949	0	0.5	0.5	0	0	0	0	0	0	2.5
32	Shanghai	(2021) Hu 73 Civil Final No.183	0	0.5	0.5	0	0	0	0	0	0	1.1
33	Shanghai	(2017) Hu 73 Civil First Instance No.208	0	0.5	0	0.5	0	0	0	0	0	1.1
34	Shanghai	(2021) Hu 73 Civil Final No.256	1	0.5	0.5	0	0	0	0.5	0	0	1.6
35	Shanghai	(2019) Hu 0104 Civil First Instance No.22387	1	0.5	0.5	0	0	0	0.5	0	0	2.3
36	Zhejiang	(2020) Zhe 01 Civil Final No.8158	1	0.5	0	0	0	0.5	0	1.5	0.5	2.3
37	Zhejiang	(2019) Zhe Civil Final No.292	0	0.5	0.5	0	0	1.5	0.5	0	0	1.8
38	Zhejiang	(2019) Zhe 10 Civil Final No.621	1	0.5	0.5	0	1	1	0.5	0	0	1.9
39	Zhejiang	(2020) Zhe Civil Final No.1117	1	0.5	0	0	1	1.5	0	0	0	2.8
40	Beijing	(2021) Jing 0102 Civil First Instance No.25480	1	0.5	0.5	0	0	1	0.5	0	0	2.2
41	Beijing	(2015) Jing IP Court Civil First Instance No.1677	1	0.5	0	0	0	1	0.5	0.5	0	1.8
42	Beijing	(2016) Jing 73 Civil First Instance No.277	1	0	0	0	0	1.5	0	0.5	0	2.4
43	Beijing	(2017) Jing Civil Final No.413	1	0.5	0	0	0	0	0	0	0	1.7
44	Beijing	(2017) Jing 73 Civil Final No.1991	1	0.5	0	0	0	1.5	0	0	0.5	1.6
45	Beijing	(2019) Jing 73 Civil First Instance No.1335	1	0.5	0.5	0.5	0	0.5	0.5	1	0	1.8

In April 2022, the Intellectual Property Division of the Beijing High Court meticulously reviewed recent civil cases involving punitive damages for intellectual property (IP). Subsequently, five representative cases of punitive damages for IP were selected, denoted as cases No. 41 to No. 45 in **Table 3.**

**Fig 5:**
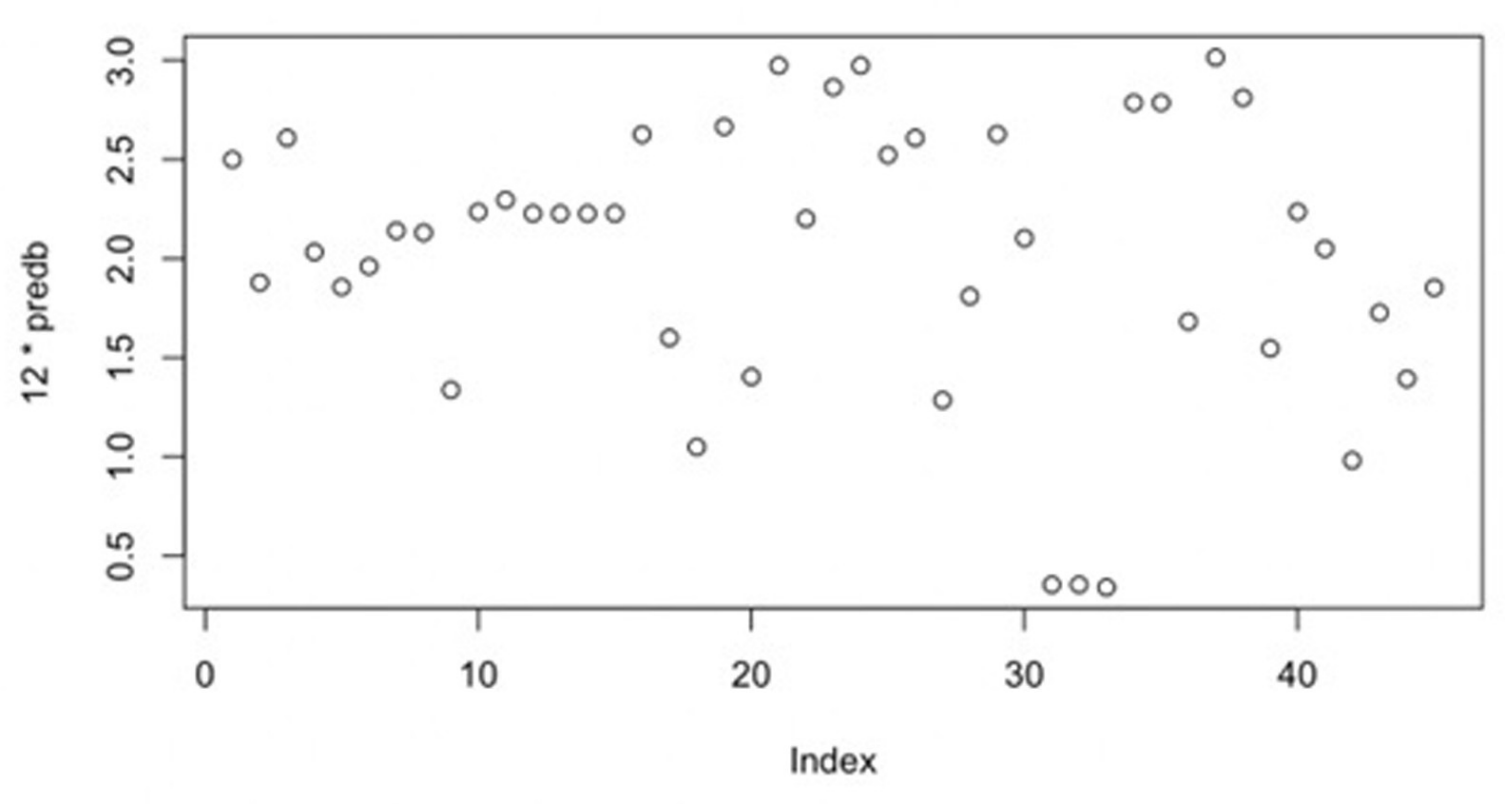
Residuals of the data validation.

**Fig 5** shows that all forecasted values inherently remain non-negative due to the correct specification of the Generalized Linear Model (GLM). This outcome is achieved independently of the estimated impacts of the individual predictors, some of which display negative point estimates. Overall, while the equation results align with the general trend of judicial judgments, in individual case assessments, if there are no "high-weighting circumstances" present, the predicted punitive damage multiplier tends to be lower than the actual judgment rendered by the court.

## 4. Discussion

Examining judicial practices extends beyond the interpretation of specific decision-making actions; it aims to unveil the systemic nature of the overall legal practice through careful observation and interpretation of specific decision-making behaviors. While qualitative research provides valuable insights into decision-making behaviors, its effectiveness can be limited by the lack of scientific rigor [22]. Conversely, legal research grounded in quantitative analysis enhances the depth and precision of conclusions. Quantitative analysis responds to legislative needs and challenges, and refines judicial theories, particularly in areas where establishing clear referee standards proves challenging [23]. In addressing complex legal scenarios where referee standards may be ambiguous, integrating artificial intelligence (AI) technology emerges as a viable option [24]. AI has already been employed to assist judges in making decisions, such as determining suspects’ release and bail status. In some instances, judges increasingly rely on AI "black boxes" to provide decision-making support [25]. Notably, China’s Zhejiang Province has developed an AI judge assistant named "Xiaozhi," capable of efficiently summarizing dispute focal points and generating judgment documents. Xiaozhi has significantly reduced average trial times by 57.8% and demonstrated a remarkable over 95% completeness rate in automatically generating adjudication documents for various legal fields, including sale and purchase contracts and cases related to dangerous driving [26].

Determining the amount of damages for intellectual property rights is a world-class problem. The punitive damages system is the sixth type of damages in China’s IPR compensation system, following "the plaintiff’s loss," "the defendant’s infringement profit," "the referable license fee," "statutory damages," and "discretionary damages." Statutory damages" and "discretionary damages" are the sixth way of calculating damages in China’s IPR system. There is a dilemma in applying the punitive damages. In judicial practice, applying punitive damages’ subjective impact is too high. Implementing statutory damages instead of punitive damages cannot provide the parties with stable judicial expectations. Existing research for qualitative analysis has not been through the research method of econometric jurisprudence on punitive damages factors for punitive damages multiplier contribution ratio quantitative research. Econometric jurisprudence is a science that uses electronic computers and other means to introduce quantitative calculation methods into the field of law to quantitatively calculate and analyze the enactment, implementation, and observance of laws, as well as legal education and jurisprudential research. This paper aims to establish the relationship between punitive damage multiplier and its influencing factors and express it in a mathematical model. However, our data sources are minimal. Although we reviewed more than 3,000 judgments with the punitive damages keyword, only 79 judgments were able to extract data. Therefore, it is worth discussing how to rely on limited sample data to conduct numerical experiments. In this paper, we have used two statistical models to verify the results, and in the future, we can also use machine learning to refine models, which is worth trying.

It is essential to note that not all cases may be suitable for AI trials. Cases with well-defined adjudication bases, such as those involving punitive damages, necessitating case-by-case comparisons, are more amenable to AI assistance. Looking ahead, integrating artificial intelligence technology, particularly large models, holds the potential for infinite expansion in the iterative process of "practice-precipitation-repractice" through machine learning. This continuous evolution promises to enhance the quality of judicial decisions.

## 5. Conclusion

In conclusion, this article first determines the factors contributing to "maliciousness" and "serious circumstances," assigning corresponding index factors to formulate a foundational equation. A comprehensive review and refinement of judicial decisions comprised a factor scale encompassing nine factors, with detailed scoring criteria for each factor serving as the foundation for judgment evaluation. These factors were then applied to annotate collected judgments, addressing numerical extraction challenges by analyzing and integrating corresponding extraction standards. Subsequently, values for each judgment’s influencing factors of the punitive damage multiplier were determined. Using statistical regression analysis, we explored the underlying patterns, proposed a conditional least squares equation, and derived the punitive damages judgment equation. We enhanced the model by transitioning to a Beta Generalized Linear Model, which led to the removal of negative coefficients. Moreover, a higher R-squared value corroborated this modification compared to the prior model. By analyzing the results from both models, we demonstrated that the extent of infringement and the reputation of intellectual property assets are critical variables. Our analysis suggests that broader infringements result in more substantial economic damages, highlighting the considerable market value and potential financial losses associated with intellectual property. Subsequent validation of the equation’s effectiveness was conducted using existing cases. Finally, the article comprehensively discussed the equation’s implications, drawing pertinent conclusions based on the findings.

## Supporting information

S1 Table (DOCX)

S2 Table (DOCX)
