# Supplementary material for: Calculating punitive damage multiplier in intellectual property cases: An empirical study and the enhanced model
Source: PLoS One. 2025 Feb 21;20(2):e0308447. doi: 10.1371/journal.pone.0308447 (PMC11844832; doi:10.1371/journal.pone.0308447)
Supplement: S1 Table — (DOCX) [file pone.0308447.s001.docx]

| IP | Aware | Repeat | Conceal | Profession | Duration | Scope | Losses | Conseq | Actual |
| --- | --- | --- | --- | --- | --- | --- | --- | --- | --- |
| 0 | 0.5 | 1 | 0 | 1 | 1.5 | 1 | 1.5 | 0 | 5 |
| 1 | 0.5 | 0 | 0 | 0 | 0 | 0 | 0 | 0 | 2 |
| 1.5 | 0.5 | 0 | 0 | 0 | 0.5 | 0.5 | 0.5 | 0.5 | 3 |
| 1 | 0.5 | 0.5 | 0 | 1 | 1 | 0 | 0 | 0.5 | 2 |
| 1.5 | 0 | 0 | 0 | 1 | 1.5 | 0.5 | 0.5 | 1 | 1 |
| 1 | 0.5 | 0 | 0 | 0 | 0.5 | 0.5 | 0.5 | 0 | 1.5 |
| 1 | 0.5 | 0 | 0 | 1 | 1 | 0 | 0.5 | 0.5 | 3 |
| 1 | 0.5 | 0 | 0 | 1 | 1.5 | 1 | 1.5 | 0.5 | 3 |
| 1 | 1 | 0.5 | 0 | 0 | 2 | 0.5 | 0 | 0.5 | 2 |
| 1 | 1 | 0 | 0.5 | 1.0 | 0.5 | 0.5 | 1.5 | 0.5 | 3 |
| 0 | 0 | 0.5 | 0 | 0 | 1.5 | 0.5 | 0 | 0 | 3.0 |
| 1 | 0.5 | 0.5 | 0 | 0 | 1.0 | 0 | 0 | 1 | 2.0 |
| 1 | 0.5 | 0.5 | 0 | 0 | 1.5 | 0 | 0 | 0.5 | 1.0 |
| 1 | 0.5 | 0.5 | 0 | 0 | 1.5 | 0.5 | 0 | 0.5 | 2.0 |
| 1 | 0.5 | 0.5 | 0 | 0 | 1.5 | 0.5 | 0 | 0.5 | 2.0 |
| 1.5 | 0.5 | 0.5 | 0 | 1 | 1.5 | 0.5 | 0 | 0 | 2.0 |
| 1 | 0.5 | 0.5 | 0 | 0 | 1.5 | 0.5 | 0 | 0 | 2.5 |
| 1.5 | 0.5 | 0.5 | 0 | 0 | 1.5 | 0.5 | 0 | 0.5 | 2.0 |
| 1 | 0.5 | 0 | 0 | 0 | 0 | 0.5 | 0 | 0.5 | 3.0 |
| 1.5 | 0.5 | 0 | 0 | 0 | 0 | 0.5 | 0 | 0 | 4.0 |
| 0.5 | 0.5 | 0.5 | 0 | 0 | 0.5 | 0.5 | 0 | 0.5 | 2.0 |
| 1 | 0.5 | 0 | 0 | 1 | 1.5 | 0.5 | 1.5 | 1 | 3.0 |
| 1 | 0.5 | 0 | 0 | 1 | 0.5 | 0.5 | 0 | 0 | 4 |
| 1 | 0.5 | 0 | 0 | 0 | 1 | 0.5 | 0 | 1 | 5.0 |
| 1 | 0.5 | 0 | 0 | 0 | 0.5 | 0.5 | 0 | 0.5 | 3.0 |
| 0.5 | 0.5 | 0 | 0 | 0 | 0.5 | 0.5 | 0 | 0.5 | 3.0 |
| 0.5 | 0.5 | 1 | 0 | 0 | 1.5 | 0.5 | 0 | 0.5 | 3.0 |
| 0.5 | 0.5 | 0 | 0 | 0 | 1.5 | 0.5 | 0 | 0.5 | 2 |
| 1 | 0.5 | 0 | 0 | 1.0 | 0.5 | 0 | 0 | 0.5 | 1.0 |
| 1 | 0.0 | 0 | 0 | 0 | 1 | 0 | 0 | 0.5 | 2 |
| 1 | 0.5 | 0.5 | 0 | 0 | 1 | 0.5 | 0 | 0 | 1.5 |
| 0 | 0.5 | 0.5 | 0 | 0 | 1 | 0.5 | 0 | 0 | 1.5 |
| 1 | 0.5 | 0 | 0 | 0 | 0.5 | 0.5 | 0 | 1 | 2 |
| 1 | 0.5 | 0.5 | 0 | 1 | 1 | 0 | 1.5 | 0.5 | 3 |
| 1 | 1.5 | 0 | 0 | 0 | 1.5 | 0 | 1.5 | 0 | 1 |
| 0 | 0.5 | 0 | 0.5 | 0 | 1.5 | 0.5 | 0.5 | 0.5 | 3 |
| 1 | 0.5 | 0 | 0 | 0 | 0 | 0.5 | 1.5 | 0.5 | 2 |
| 1 | 0.5 | 1.5 | 0 | 1 | 1.5 | 0.5 | 0 | 0.5 | 2.7 |
| 1 | 0.5 | 0.5 | 0 | 0 | 1 | 0.5 | 1.5 | 0.5 | 1.7 |
| 1 | 0.5 | 0.0 | 0 | 1 | 1.0 | 0.5 | 0 | 0.5 | 2.0 |
| 1.5 | 0.5 | 0.0 | 0 | 1 | 1 | 0.5 | 0 | 0 | 2.0 |
| 1.5 | 1.0 | 0 | 0 | 0 | 1.5 | 0.5 | 1.5 | 0.5 | 1.0 |
| 1 | 0.5 | 0.5 | 0 | 0 | 1.5 | 0 | 0.5 | 0.5 | 1.7 |
| 1.5 | 0.5 | 0.5 | 0 | 1 | 1.5 | 0.5 | 0.5 | 0.5 | 3.0 |
| 1.5 | 0.5 | 0 | 0 | 0 | 0.5 | 0.5 | 1.5 | 0 | 2.0 |
| 0 | 0.5 | 0.5 | 0 | 0 | 1 | 0.5 | 0 | 0 | 1.5 |
| 1 | 0.5 | 0 | 0 | 0 | 1.5 | 0.5 | 1.5 | 0.5 | 2.0 |
| 1 | 0.5 | 0 | 0 | 0 | 1.5 | 0.5 | 0 | 0 | 1.0 |
| 0 | 0.5 | 0.5 | 0 | 0 | 1 | 0.5 | 0.0 | 0.0 | 1 |
| 0 | 0.5 | 0.0 | 0 | 0 | 1 | 0.5 | 0.0 | 0.0 | 1.5 |
| 1 | 0.5 | 0 | 0 | 0 | 0 | 0.0 | 0 | 0 | 2.0 |
| 1.5 | 0.5 | 0 | 0 | 0 | 0 | 0.5 | 0 | 0 | 5.0 |
| 1.5 | 0.5 | 0 | 0 | 0 | 0 | 0.5 | 0 | 0 | 5.0 |
| 1.5 | 0.5 | 0 | 0 | 0 | 0 | 0.5 | 0 | 0 | 5.0 |
| 1 | 0.5 | 0 | 0 | 0 | 0.0 | 0.5 | 0 | 1.0 | 5.0 |
| 1 | 0.5 | 0.5 | 0 | 0 | 1.5 | 0.5 | 0 | 0 | 3.0 |
| 1 | 0.5 | 0 | 0 | 1.0 | 0.5 | 0.5 | 0.5 | 0.0 | 3 |
| 0 | 0.5 | 0 | 0 | 1.0 | 1.5 | 0.5 | 1.5 | 0 | 5.0 |
| 1 | 0.5 | 0.5 | 0 | 0 | 0.5 | 0.5 | 0.5 | 0.5 | 2 |
| 1.5 | 1 | 1.5 | 0 | 1.0 | 1.5 | 0.5 | 0.5 | 0 | 3.0 |
| 1 | 0.5 | 1.5 | 0 | 0 | 1.5 | 0.0 | 0 | 1.0 | 1.0 |
| 0 | 0.5 | 0.5 | 0 | 0.0 | 1.0 | 0.5 | 0.0 | 0.0 | 1 |
| 0 | 0.5 | 0.5 | 0 | 0 | 0 | 0.5 | 0 | 0 | 1 |
| 0 | 0.5 | 0.5 | 0 | 0.0 | 0.5 | 0.5 | 0.0 | 0.0 | 1 |
| 0 | 0.5 | 0.5 | 0 | 0.0 | 0.5 | 0.5 | 0.0 | 0.0 | 1 |
| 0 | 0.5 | 0.5 | 0 | 0 | 0.5 | 0.5 | 0.0 | 0.0 | 1 |
| 0 | 0.5 | 0.5 | 0 | 0 | 0.5 | 0.5 | 0.0 | 0.0 | 1 |
| 1 | 0 | 0.5 | 0 | 1 | 0 | 0.5 | 0 | 0 | 1.0 |
| 1 | 0.5 | 1 | 0 | 1 | 0.5 | 0 | 0.5 | 0.5 | 2.0 |
| 0 | 0.5 | 0.0 | 0 | 0 | 1.0 | 1.0 | 0.5 | 0 | 4.0 |
| 1 | 0.5 | 0.5 | 0 | 0 | 0.5 | 0.5 | 0 | 0 | 5 |
| 1.5 | 0.5 | 0.5 | 0 | 1 | 1.5 | 1 | 1.5 | 1.0 | 4.0 |
| 1 | 0.5 | 0.5 | 0 | 1 | 1.5 | 0.5 | 0 | 0.5 | 3.0 |
| 1 | 0.5 | 1 | 0 | 1 | 1.5 | 0.5 | 0 | 0 | 3.5 |
| 1.5 | 0.5 | 0.5 | 0 | 1 | 1.5 | 0 | 1.5 | 0.5 | 4.0 |
| 1 | 0.5 | 0.5 | 0 | 0 | 1.5 | 0.5 | 0 | 0.5 | 2.0 |
| 0.5 | 0.5 | 0.5 | 0 | 0 | 1.5 | 0.5 | 0 | 0.5 | 3.0 |
| 1 | 0.5 | 0 | 0 | 0 | 0.5 | 0.5 | 0.5 | 0 | 1.5 |
| 1.5 | 0.5 | 1.5 | 0 | 0.0 | 1.5 | 0.5 | 0.0 | 0.0 | 3 |
