# Supplementary material for: Calculating punitive damage multiplier in intellectual property cases: An empirical study and the enhanced model
Source: PLoS One. 2025 Feb 21;20(2):e0308447. doi: 10.1371/journal.pone.0308447 (PMC11844832; doi:10.1371/journal.pone.0308447)
Supplement: S2 Table — (DOCX) [file pone.0308447.s002.docx]

| **IP** | **Aware** | **Repeat** | Conceal | Profession | Duration | Scope | Losses | Conseq | Actual |
| --- | --- | --- | --- | --- | --- | --- | --- | --- | --- |
| 1 | 0.5 | 0.5 | 0 | 0 | 0.5 | 0.5 | 0 | 0 | 2.3 |
| 0.5 | 0.5 | 0.5 | 0 | 0 | 1.5 | 0 | 0 | 0.5 | 1.6 |
|  | 0.5 | 0 | 0 | 0 | 0 | 0.5 | 0 | 0 | 2.4 |
| 1 | 0.5 | 0 | 0 | 1 | 1.5 | 0 | 0.5 | 1 | 2.2 |
| 1 | 0.5 | 0 | 0 | 0 | 1.5 | 0.5 | 0 | 0 | 2.3 |
| 1 | 0.5 | 0 | 0 | 0 | 0 | 0 | 0.5 | 0.5 | 2.4 |
| 1 | 0.5 | 0 | 0 | 0 | 1.5 | 0.5 | 0 | 0.5 | 1.7 |
| 1 | 0 | 0.5 | 0 | 0 | 0.5 | 0.5 | 0 | 0 | 2.4 |
| 0 | 0 |  | 0 | 0 | 1.5 | 0 | 0 | 0 | 2.3 |
| 1 | 0.5 | 0.5 | 0 | 0 |  | 0.5 | 0 | 0 | 1.3 |
| 0 | 0.5 | 0 | 0 | 0 | 1 | 0.5 | 0 | 0 | 2.4 |
| 1 | 0 | 0 | 0 | 0 | 0 | 0.5 | 0 | 0 | 1.8 |
| 1 | 0 | 0 | 0 | 0 | 0 | 0.5 | 0 | 0 | 2.2 |
| 1 | 0 | 0 | 0 | 0 | 0 | 0.5 | 0 | 0 | 2.2 |
|  | 0 | 0 | 0 | 0 | 0 | 0.5 | 0 | 0 | 2.2 |
|  | 0.5 | 1.5 | 0 | 0 | 1.5 | 0.5 | 0 | 0.5 | 2.2 |
| 0.5 | 0 | 0 | 0 | 0 | 0 | 0.5 | 0.5 |  | 2.6 |
| 0.5 | 0 | 0 | 0.5 | 0 | 0 | 0.5 | 0 | 0.5 | 2.1 |
| 0.5 | 0.5 | 0 | 0 | 0 | 1.5 | 0.5 | 0 | 0 | 2.5 |
| 0.5 | 0 | 0 | 0 | 0 | 0 | 0.5 | 0 | 0.5 | 2.1 |
| 1 | 0.5 | 0 | 0 | 0 | 0 | 0.5 | 0 | 0.5 | 2 |
|  | 0.5 | 0 | 0 | 1 | 0 | 0 | 0 | 0 | 2.3 |
| 1 | 1 | 1.5 | 0 | 0 | 0 | 0 | 0 | 0.5 | 2 |
| 1 | 0.5 | 0 | 0 | 0 | 0 | 0.5 | 0 | 0.5 | 1.8 |
| 1 | 0.5 | 0.5 | 0 | 1 | 1.5 | 0.5 | 0 | 0 | 2.3 |
| 1 | 0.5 | 0 | 0 | 0 | 0 | 0.5 | 0 | 0 | 2.9 |
| 1 | 0 | 0 | 1 | 0.5 | 0.5 | 0.5 | 0 | 0 | 2.2 |
| 1.5 | 0.5 | 1.5 | 0 | 0 | 1.5 | 0 | 1.5 | 0.5 | 3.5 |
|  | 0.5 | 0.5 | 0 | 0 | 1.5 | 0.5 | 0 | 1 | 2.3 |
| 1 | 0.5 | 1 | 0 | 0 | 1.5 | 0.5 | 0.5 | 0 | 2.5 |
| 0 | 0.5 | 0.5 | 0 | 0 | 0 | 0 | 0 | 0 | 2.5 |
| 0 | 0.5 | 0.5 | 0 | 0 | 0 | 0 | 0 | 0 | 1.1 |
| 0 | 0.5 | 0 | 0.5 | 0 | 0 | 0 | 0 | 0 | 1.1 |
| 1 | 0.5 | 0.5 | 0 | 0 | 0 | 0.5 | 0 | 0 | 1.6 |
| 1 | 0.5 | 0.5 | 0 | 0 | 0 | 0.5 | 0 | 0 | 2.3 |
| 1 | 0.5 | 0 | 0 | 0 | 0.5 | 0 | 1.5 | 0.5 | 2.3 |
| 0 | 0.5 | 0.5 | 0 | 0 | 1.5 | 0.5 | 0 | 0 | 1.8 |
| 1 | 0.5 | 0.5 | 0 | 1 |  | 0.5 | 0 | 0 | 1.9 |
| 1 | 0.5 | 0 | 0 | 1 | 1.5 | 0 | 0 | 0 | 2.8 |
| 1 | 0.5 | 0.5 | 0 | 0 |  | 0.5 | 0 | 0 | 2.2 |
|  | 0.5 | 0 | 0 | 0 | 1 | 0.5 | 0.5 | 0 | 1.8 |
|  | 0 | 0 | 0 | 0 | 1.5 | 0 | 0.5 | 0 | 2.4 |
| 1 | 0.5 | 0 | 0 | 0 | 0 | 0 | 0 | 0 | 1.7 |
| 1 | 0.5 | 0 | 0 | 0 | 1.5 | 0 | 0 | 0.5 | 1.6 |
| 1 | 0.5 | 0.5 | 0.5 | 0 | 0.5 | 0.5 | 1 | 0 | 1.8 |
